# Continuous and Flash Glucose Monitoring in Adults at Risk of Type 2 Diabetes: A Scoping Review

**DOI:** 10.1177/19322968251315497

**Published:** 2025-02-17

**Authors:** Prince Amoh, David Broom, Ioannis Kyrou, Samuel Nartey, Anna Paul, Dale Esliger, Maxine Whelan

**Affiliations:** 1 Coventry University, Coventry, UK; 2 University Hospitals Coventry and Warwickshire NHS Trust, Coventry, UK; 3 University of Warwick, Coventry, UK; 4 Aston University, Birmingham, UK; 5 University of Derby, Derby, UK; 6 Kwame Nkrumah University of Science and Technology, Kumasi, Ghana; 7 School of Sport, Exercise and Health Sciences, Loughborough University, Loughborough, UK

**Keywords:** continuous glucose monitoring, flash glucose monitoring, type 2 diabetes mellitus, dietary and exercise interventions, diabetes prevention, glucose control, insulin resistance, obesity, health-related behaviors

## Abstract

**Background:**

Continuous glucose monitoring (CGM) and flash glucose monitoring (FGM) are widely used in diabetes management and increasingly being considered for type 2 diabetes mellitus (T2DM) prevention. This scoping review aims to summarize the literature published to date on CGM and FGM use in adults at risk of T2DM.

**Methods:**

A systematic search of four databases (CINAHL, PsycINFO, MEDLINE, Cochrane Library) was conducted, covering studies from 1985 to 2024. Eligible studies used CGM or FGM in interventional settings targeting adults at risk of T2DM. Rayyan software facilitated article screening, and the Johns Hopkins Evidence-Based Practice tool assessed study quality.

**Results:**

From 13 644 articles, 12 studies were included, reporting on 1144 participants (353 at-risk, mean age 47 ± 12.8 years) across eight countries. Ten studies employed FGM, focusing on health-related behaviors (diet, physical activity, or both). Significant improvements in glucose control and anthropometrics were reported in 75% and 50% of the studies, respectively, along with reductions in glycated hemoglobin, fasting glucose, and insulin resistance. Seven studies used qualitative methods, with recurrent themes including perceived benefits and motivators for behavior change and acceptability and feasibility of device use. Three studies were rated as “high” level and scored a “B” for evidence quality, while the remaining studies were lower for both level and evidence quality.

**Conclusions:**

Existing published studies deploying glucose monitoring technologies show promise in supporting interventions aimed at preventing T2DM in at-risk adults. Further robust studies are required to confirm the long-term acceptability and efficacy of these technologies.

## Introduction

Type 2 diabetes mellitus (T2DM) is among the top ten leading causes of morbidity and mortality worldwide, with an estimated 463 million people living with this condition in 2019, rising to 10.2% of the global population by 2030.^1,2^ Major risk factors for the development of T2DM include obesity, physical inactivity, unhealthy dietary habits, and genetic predisposition, with obesity and sedentary lifestyle as significant contributors in accelerating disease onset.^3^ Type 2 diabetes mellitus can often be prevented through early intervention targeting weight loss, particularly in individuals identified as being at high risk (such as people with prediabetes or metabolic syndrome).^4^, ^5^ To support preventative strategies, innovative glucose monitoring technologies, either continuous glucose monitoring (CGM) or flash glucose monitoring (FGM) in nature, are being increasingly utilized.^6^ Providing near real-time feedback to users about glucose concentration, such technologies have been widely explored as part of diabetes management, particularly for individuals requiring intensive insulin therapy.^7,8^

Continuous glucose monitoring and FGM have demonstrated efficacy in improving glycemic control and reducing hypoglycemic events in patients with type 1 diabetes mellitus (T1DM) and insulin-treated T2DM.^9^ However, recent studies have suggested that CGM and FGM could also play a significant, yet perhaps controversial, role in the prevention of T2DM. This technology may allow users to gain a better understanding of how health-related behaviors, such as diet, physical activity, and other lifestyle behaviors, impact on glucose control throughout the day over several days. As such, these technologies may play a beneficial role in motivating positive changes in these behaviors which could lead to reduced risk of developing T2DM.^10^

Despite this potential, the use of CGM and FGM in people without diabetes, particularly those at risk of developing T2DM, remains unclear. Thus, there is a need for a systematic identification and exploration of the relevant existing literature to map and critically review the available evidence and identify gaps in this field to be addressed by future research. Accordingly, this scoping review aims to identify and critically review studies deploying CGM and FGM technologies in an interventional capacity in adults at risk of T2DM. By comprehensively summarizing the current state of research, this review contributes to the current understanding of how such technologies may support T2DM prevention in at-risk populations and highlights recommendations for future research in this field.

## Methods

The present scoping review is reported in accordance with the PRISMA (Preferred Reporting Items for Systematic Reviews and Meta-Analyses) extension for scoping reviews.^11^

### Eligibility Criteria

The eligibility criteria for this scoping review were developed using the Population, Concept, and Context (PCC) framework, in accordance with the Joanna Briggs Institute (JBI) guidelines.^12^

Population: Studies involving adults without a known diagnosis of T2DM but considered at risk of developing the condition (either due to blood test results, risk score or known risk factors such as having a body mass index (BMI) in the overweight or obesity range). Studies reporting data on combined populations (adults without and with a known diagnosis of T2DM) were included.

Concept: Studies utilizing CGM or FGM technologies in an interventional capacity.

Context: Studies intending to improve glucose management or reduce the risk of T2DM (ie, intervention-based).

A search was performed in March 2024 to identify if any existing scoping review had been conducted on this topic and none were identified (Table 1).

**Table 1.** Search for Existing Reviews.

“scoping review” AND (“continuous glucose monitor” OR “continuous glucose monitoring” OR “flash glucose monitor” OR “flash glucose monitoring”)

### Search Strategy

A systematic search was conducted on May 1, 2024 across four databases: CINAHL, PsycINFO, MEDLINE, and the Cochrane Library. The search was designed to capture published literature from 1985 to 2024 and was collaboratively developed by the lead reviewer (PA), an experienced information scientist (SD), and the wider research team (MW, DB, IK). Search terms were developed using both controlled vocabulary (eg, MeSH terms) and free-text terms aligning to the PCC framework to ensure a thorough retrieval of relevant studies. The search strategy tailored to each database is detailed in Table 2. Reference lists of included studies were also searched for further potentially eligible articles.

**Table 2.** Search Strategy.

(“continuous glucose monitoring” OR “continuous glucose monitor” OR “cgm” OR “flash glucose monitor” OR “flash glucose monitoring” OR “freestyle libre” OR “dexcom” OR “simplera” OR “medtronic” OR “guardian” OR “eversense” OR “self monitoring technologies” OR “self-monitoring technologies”) AND (“people without diabetes” OR “persons without diabetes” OR “nondiabetic” OR “non-diabetic” OR “healthy adults” OR “healthy individuals” OR “adults without diabetes” OR “abnormal glucose tolerance” OR “impaired glucose tolerance” OR “prediabetes” OR “pre-diabetes” OR “prediabetic” OR “prediabetics” OR athlet* OR sport* OR exercis* OR “adult” OR “adults” OR normoglycaemia* OR normoglyce* OR “normal glucose tolerance” OR “normal glucose”)

### Selection of Sources of Evidence

After conducting the search, retrieved references were exported to EndNote for de-duplication and later imported into the Rayyan systematic review platform for screening.^13^ Title and abstract screening were conducted by PA and two other reviewers (AP and SN; 100% double screened). Full-text screening was completed by PA, with 50% of the articles second-screened by SN. Conflicts during the screening process (19 after title/abstract screening and 7 after full-text screening) were easily resolved through discussions with the reviewers without a need for the involvement of the wider research team.

### Data Extraction and Charting

Data were independently extracted from the included studies using a predefined data extraction form based on the JBI framework.^12^ An initial form was piloted with three of the included studies before refinements were made. In the final form, the extracted data included lead author, publication year, study location, study design, sample size and participant characteristics, intervention duration, intervention components, and outcome measures (including quantitative and qualitative data). For studies that incorporated qualitative methods, key findings reported in the studies were extracted and reviewed multiple times to identify recurring patterns across studies. Data were then grouped into broad themes and described. Extracted data were presented narratively alongside tables to summarize key findings.

### Critical Appraisal

Although not mandatory for a scoping review, the methodological quality of included studies was appraised using the Johns Hopkins Nursing Evidence-Based Practice (JHEBP) tool for research evidence to assess evidence level and quality of research.^14^ Classification and scores were based on the level of evidence (level I, II, or III) and quality of research (high [A], good [B], or low quality [C]) reported.

## Results

### Search Results

**Figure 1.**
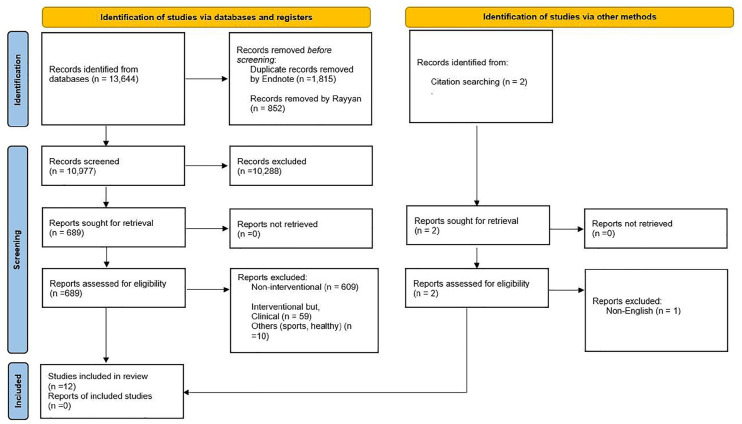
PRISMA chart.

The initial search across the four searched databases yielded 13 644 articles (Figure 1). After de-duplication, 10 977 studies remained for title and abstract screening. The full screening process resulted in the inclusion of 11 eligible studies. One additional study was identified through a primary reference list search, bringing the total to 12 included studies.

### Characteristics of the Included Studies

#### Location, timing, and duration

The earliest study was published in 2016,^15^ with most of the studies (*n* = 10) published between 2020 and 2023. The included studies represented research conducted in eight countries and across four continents, with the United States being the most frequent study location (*n* = 4). Study duration ranged from ten days to 52 weeks, with a median duration of eight weeks (interquartile range: 6-16 weeks).

#### Study design

Four of the included studies were randomized controlled trials (RCTs),^16-19^ with the remaining studies being randomized feasibility trials (*n* = 3),^20-22^ pilot interventional and feasibility studies (*n* = 4),^15,22-24^ observational study (*n* = 1),^25^ or a randomized controlled crossover design (*n* = 1).^26^ Further details of the study designs are summarized in Table 3.

**Table 3.** Pertinent Study Characteristics of the Included Studies.

Author, country	Purpose of study	Study design	Total number of participants	Number of participants that used CGM	Number of at-risk participants that used CGM	Study duration	Critical appraisal
Bailey et al,^15^ Canada	To demonstrate the potential of using CGM to foster self-monitoring and increase exercise adherence in individuals with prediabetes and T2DM	Quasi-experimental feasibility study	13	7	*	8 weeks	IIIB
Whelan et al,^20^ England	To determine the effectiveness of using glucose and physical activity self-monitoring technologies for lifestyle changes in individuals at moderate to high risk of developing T2DM	Randomized feasibility trial	45	45	45	6 weeks	IIB
Liao et al,^23^ United States	To determine the acceptability of a physical activity intervention that incorporated the use of CGMs in sedentary adults with overweightness and obesity without diabetes, and to evaluate the changes in exercise motivation	Pilot interventional study	19	19	19	10 days	IIIB
Röhling et al,^16^ Germany	To assess the effectiveness of the Low-Insulin-Method integrated into the program for weight reduction and long-term weight maintenance in overweight or obese individuals	Randomized controlled trial	30	30	30	52 weeks	IB
Yost et al,^24^ United States	To determine the satisfaction and feasibility of an intervention combining CGM use and low-carbohydrate diet coaching in patients with prediabetes to drive dietary behavior change	Pilot feasibility study	15	15	15	22 days	IIIB
Jospe et al,^21^ New Zealand	To compare the effect of using CGM versus traditional fingerpick glucose monitoring on adherence to a hunger training program, weight loss, body composition, HbA1c, eating behaviors, and psychological health	Randomized feasibility trial	40	20	4	6 months	IIB
Dehghani Zahedani et al,^25^ United States	To examine whether the use of a digital tracker combined with CGM can improve glucose regulation in healthy, adults with prediabetes and those with non–insulin-treated T2DM	Observational trial	665	25	25	10 days	IIB
Schembre et al,^22^ United States	To determine the feasibility of adding hunger training using glucose monitoring to the Diabetes Prevention Program and explore effects on weight, metabolic, and breast cancer risk biomarkers	Randomized feasibility study	50	27	27	16 weeks	IIB
Chekima et al,^17^ Malaysia	To measure the effects of integrating real-time CGM into a low glycemic index and glycemic load dietary intervention on dietary intake, body composition, and specific metabolic parameters among overweight and obese young adults	Randomized controlled trial	40	20	20	8 weeks	IB
Chekima et al,^26^ Malaysia	To determine the glycemic index of three varieties of rice-based mixed meals and their effects on glycemic variability, 24-hour mean glucose levels, and rice variety preferences among overweight and obese young adults using real-time CGM	Randomized crossover study	14	14	14	12 days	IIB
Ahn et al,^18^ Republic of Korea	To evaluate the effectiveness of dietary coaching combined with CGM in patients with diabetes or prediabetes to improve their behavioral skills and health outcomes	Randomized controlled trial	45	45	*	4 weeks	IIB
Kitazawa et al,^19^ Japan	To investigate the effectiveness of a lifestyle intervention program combining lifestyle coaching via a smartphone application augmented by intermittently scanned CGM for glycemic control and body weight reduction in people at risk of T2DM	Randomized controlled trial	168	86	86	12 weeks	IB

Abbreviations: CGM, continuous glucose monitor; HbA1c, hemoglobin A1c; T2DM, type 2 diabetes mellitus.

* Not specified.

#### Age and number of participants

The total number of participants reported across all included studies was 1144. Participant sample sizes varied substantially, ranging from 13^15^ to 665^25^ in a single study. The mean age of participants also varied, with the youngest reported average age being 22.8 years^26^ and the oldest 62.23 years.^15^ On average, across all 12 studies, the mean age of participants was 47.01 years (standard deviation: 12.83). Further details on the age and number of participants for each included study are summarized in Table 4.

**Table 4.** Participants Characteristics of the Included Eligible Studies.

Author, country	Age (mean ± SD)	Sex distribution (% women)	At-risk/diabetes status of participants	Ethnicity	Education status
Bailey et al,^15^ Canada	62.23 ± 6.66	76.9%	Prediabetes, T2DM	Mainly white (approximately 77%)	76.9% had post-secondary education
Whelan et al,^20^ England	56 ± 9.00	60.0%	Moderate to high risk of developing T2DM	Mainly white British (88.9%)	35% had postgraduate degrees, 53% had above secondary-level education
Liao et al,^23^ United States	46.5 ± 8.32	83.3%	Overweight and obesity (T2DM included)	*	No report but participants are hospital staff, likely to have some degree of education
Röhling et al,^16^ Germany	41.5 ± 8.40	84.2%	Overweight and obesity (T2DM excluded)	47.3% African American, 15.7% Asian, 15.7% Hispanic white, 15.7% non–Hispanic white	84.2% had at least a college/technical-level degree
Yost et al,^24^ United States	54.5 ± 9.10	66.7%	Prediabetes	73.3% white, 26.7% African American	100% completed high school; 53% had a bachelor’s degree
Jospe et al,^21^ New Zealand	45.35 ± 12.88	55%	Obesity (T2DM not mentioned as exclusion criteria)	*	55.5% had a university degree
Dehghani Zahedani et al,^25^ United States	35.8 ± 7.70		Prediabetes, T2DM, healthy	12.5% white Caucasian, 12.5% black American, 5% Asian American	*
Schembre et al,^22^ United States	60.33 ± 6.00	100%	Overweight and obesity (T2DM excluded)	82% white, 16% black	88% had at least college education
Chekima et al,^17^ Malaysia	26.4 ± 5.30	57.5%	Overweight and obesity (T2DM excluded)	*	*
Chekima et al,^26^ Malaysia	22.8 ± 4.60	35.7%	Overweight and obesity (T2DM excluded)	*	*
Ahn et al,^18^ Republic of Korea	48.45 ± 10.48	62.2%	Prediabetes, T2DM	*	*
Kitazawa et al,^19^ Japan	48.31 ± 8.19	19.6%	High risk of developing T2DM	*	*

Abbreviation: T2DM, type 2 diabetes mellitus.

* Not reported.

#### Diabetes status of participants

Out of the 1144 participants across the included studies that used CGM/FGM, 353 adults were considered at risk of developing T2DM. Half of the studies (*n* = 6) focused on individuals who were overweight or had obesity,^16,17,21-23,26^ specifically excluding individuals with T2DM in five of these studies.^16,17,21,22,26^ Three studies (25%) involved populations with mixed diabetes status, including both prediabetes and T2DM.^15,18,25^ Three studies (25%) recruited participants with prediabetes^15,18,24^ and the remaining studies (*n* = 2) focused on moderate- to high-risk populations^19,20^ without specifying diabetes status^19,20^ (Table 4).

#### Sex distribution

The sex distribution across the included studies varied (Table 4), with a higher proportion of women participants in most (*n* = 8)^15-18,20,21,23,24^ studies. In one study,^22^ all participants were women, while men were the majority in two other studies.^19,26^ One study did not report the sex distribution of participants.^25^

#### Ethnicity

The reporting of participant ethnicity varied across the included studies, with 4 of the 12 studies^16,22,24,25^ providing specific data on racial/ethnic backgrounds, while the remaining 8 studies either did not report^17-19,21,23,26^ this information or offered only partial details^15,20^ (Table 4).

In one study,^15^ most participants were white (two study arms having 71% and 83% of participants identified as white). Another study reported that 88.9% of the participants were white British,^20^ while two other studies^22,24^ had >70% white participants. Two studies^16,25^ included more ethnically diverse populations, with one^16^ reporting participants of African American, Asian, Hispanic and non–Hispanic white, and multiracial backgrounds.^4^

#### Education level

Education level was reported in six (50%) studies,^15,16,20-22,24^ with most participants in these studies reporting post-secondary education (Table 4). In most cases, over two thirds of participants held higher education qualifications, with two studies reporting that at least 84.2% of participants had a college or university degree.^16,22^

### Intervention Components

All but one study employed FGM from Abbott (ie, Freestyle Libre) with participants having to scan their sensors to obtain glucose readings. In the remaining study,^15^ the Medtronic Guardian was used.

The other intervention components employed in parallel to the glucose monitoring technologies in the included studies targeted changes in physical activity, diet-related behaviors, or a combination of both (Table 5). Three studies^15,20,23^ (25%) focused exclusively on exercise or physical activity, often supplemented with some form of education (group-mediated cognitive-behavioral interventions,^15^ one-on-one counseling)^23^ and/or additional tools, such as physical activity monitors.^20,23^ Five studies^17,21,22,24,26^ solely targeted diet-related behaviors, including low-carbohydrate diet education,^24^ hunger training,^22^ and education on low glycemic index/load diets.^17^ One study included specific dietary components, such as meal preferences based on glycemic index values.^26^ Four studies^16,18,19,25^ employed a multi-component approach, with one of these studies^16^ combining three components (group-based seminars, telemedicine coaching, and monitoring devices such as pedometers and heart rate monitors) in addition to CGM and low-carbohydrate nutrition. Two studies^19,25^ also integrated a mobile application to provide education and track food intake, physical activity, heart rate, and glucose concentrations.

**Table 5.** Components of the Interventions of the Included Eligible Studies.

Author, country	Brand of the glucose monitoring technology	Dietary components	Physical activity components	Education components	Other
Bailey et al,^15^ Canada	Medtronic Guardian		Exercise adherence	Self-monitoring behavior	Group-mediated cognitive behavioral intervention
Whelan et al,^20^ England	Abbott Freestyle Libre		Physical activity monitor (Fitbit Charge 2)		
Liao et al,^23^ United States	Abbott Freestyle Libre		Physical activity monitor (Fitbit Alta HR)	One-on-one counseling	
Röhling et al,^16^ Germany	Abbott Freestyle Libre	Low-carbohydrate nutrition using formula diets	Pedometer (smartLAB walk P+)	Group-based seminars Telemedicine coaching	
Yost et al,^24^ United States	Abbott Freestyle Libre	Low-carbohydrate diet coaching		One-on-one coaching Access to *Always Hungry* book	
Jospe et al,^21^ New Zealand	Abbott Freestyle Libre	Hunger training		General guidance and support on hunger training	
Dehghani Zahedani et al,^25^ United States	Abbott Freestyle Libre	Formula diets to generate glycemic responses to standardized nutrient challenges	Heart rate monitor (MiBand 3 or Garmin watch)		Sugar AI mobile application
Schembre et al,^22^ United States	Abbott Freestyle Libre	Hunger training for intervention group		Diabetes prevention program sessions (covers both diet and exercise)	
Chekima et al,^17^ Malaysia	Abbott Freestyle Libre			Education on low glycemic index and load diets	
Chekima et al,^26^ Malaysia	*	Provision of three rice-based mixed meals			
Ahn et al,^18^ Republic of Korea	Abbott Freestyle Libre			Non-contact dietary coaching (both as a group and individually)	Personal and social motivation interventions
Kitazawa et al,^19^ Japan	Abbott Freestyle Libre			Access to educational resources through the Health2Sync app	Health2Sync mobile application

*Not specified.

### Quantitative Outcomes Reported

Outcomes measured across the included studies were categorized into five main types: (1) anthropometric measures, (2) metabolic and biochemical markers of glucose control, (3) other metabolic and biochemical markers, (4) behavioral change and patient-reported outcome measures (PROMs), and (5) other (eg, feasibility and acceptability of the technology; Table 6). Study-level findings for the various outcomes are reported in Table 7.

**Table 6.** Quantitative Outcomes Measured In the Included Studies.

Author, country	Anthropometrics	Biochemical/metabolic markers of glucose control	Other biochemical/metabolic markers	PROMs and behavioral outcomes	Other
Bailey et al,^15^ Canada	BMI, waist circumference			Self-efficacy, self-monitoring and goal setting, physical activity behavior, health-related quality of life	
Whelan et al,^20^ England					Usage of CGM, feasibility, acceptability
Liao et al,^23^ United States				Exercise motivation (BREQ-2, URICA 2)	Acceptability of CGM use
Röhling et al,^16^ Germany	BMI, body mass	HbA1c, fasting insulin, fasting glucose, HOMA-IR	Cardio-metabolic risk factors (FRS, criteria of metabolic syndrome), lipid profile, blood pressure	Eating behavior (TFEQ—Cognitive Control, Suggestibility, Hunger), quality of life (SF-12—Physical health, Mental Health), physical activity (FFKA—sports/week, physical activity/week)	
Yost et al,^24^ United States	Body mass	eHbA1c, average daily glucose, % of time spent in hyperglycemia		Risk perception for developing diabetes, Modified Weight Loss Readiness Test II	Satisfaction, feasibility, acceptability
Jospe et al,^21^ New Zealand	Body mass, BMI	HbA1c	Lean and fat mass index		Adherence to hunger training (number of glucose measurements, booklet entry, lapses, satiety)
Dehghani Zahedani et al,^25^ United States		Time in range, fasting glucose levels			
Schembre et al,^22^ United States	Body mass	HOMA-IR, fasting glucose, fasting insulin,	Lipid profile, breast cancer biomarkers (CRP, Adiponectin, IGF-1, IGF-2, IGFBP-2)		Feasibility of hunger training (accrual goal >50%, retention >80%, average protocol adherence >75%); service satisfaction, acceptability of hunger training (difficulty of eating according to glucose levels, helpfulness of wearing CGM)
Chekima et al,^17^ Malaysia	Body mass, BMI	Glycemic index (calculated from IAUC of glucose response), fasting plasma glucose, HbA1c, plasma insulin, HOMA-IR	Fat mass, muscle mass, lipid profile	Physical activity (MET-min/week)	Glycemic index and glycemic knowledge, dietary intake (total energy, carbohydrate, protein, and fat intake)
Chekima et al,^26^ Malaysia		Glycemic variability (% CV), mean glucose level, glucose target range			Glycemic index (calculated from IAUC of glucose response); rice meal preference
Ahn et al,^18^ Republic of Korea	BMI, waist circumference, high circumference	HbA1c, HOMA-IR	Apolipoprotein A to B ratio	Eating self-efficacy, depression, insomnia	
Kitazawa et al,^19^ Japan	BMI, waist circumference	HbA1c, time in range, TAR, TBR, CV, MAGE	Blood pressure		Nutrient intake

Abbreviations: BMI, body mass index; PROM, patient-reported outcome measures; T2DM, type 2 diabetes mellitus; HOMA-IR, homeostatic model assessment for insulin resistance; BREQ-2, The Behavioral Regulation in Exercise Questionnaire-2; URICA-2, The Exercise Stages of Change—Continuous Measure; SF-12, Short Form 12 Questionnaire; TFEQ, Three-Factor Eating Questionnaire; HbA1c, glycated hemoglobin; FFkA, Freiburger Questionnaire for Physical Activity; IGF, insulin-like growth factor; IGFBP, insulin-like growth factor binding protein; IAUC, incremental area under the curve; MET, metabolic equivalent of task; TAR, time above range; TBR, time below range; MAGE, mean amplitude of glucose excursion.

**Table 7.** Quantitative Findings From Included Eligible Studies.

Author, country	Anthropometrics	Biochemical/metabolic markers of glucose control	Other biochemical/metabolic markers	PROM and behavioral outcomes	Other
Bailey et al,^15^ Canada	Decrease in waist circumference			Increase in self-efficacy and monitoring; increase in HRQL	
Whelan et al,^20^ England					Several glucose sensors displaced
Liao et al,^23^ United States	Weight loss; BMI reduction	Reduction in HbA1c Reduction in fasting insulin		Reduced CVD risk as assessed by the Framingham Risk Score	
Röhling et al,^16^ Germany				Decrease in amotivation; increase in intrinsic regulation; decrease in precontemplation and increase in the action stage for exercise motivation	100% of participants agree with statement regarding CGM usability and confidence in its information; 95% agree with statements regarding relevance, convenience, value, and recommending the technology; 84% found the technology to be motivating; 26% expressed concerns about privacy
Yost et al,^24^ United States	Significant weight loss but not different with group that did not use CGM				
Jospe et al,^21^ New Zealand	Weight loss	Reduction in HbA1c; Decrease in % time spent in hyperglycemia and average glucose		Increase in motivation to lose weight; reduction in estimated risk of developing diabetes	
Dehghani Zahedani et al,^25^ United States		Improvement of TIR over ten days			
Schembre et al,^22^ United States	Significant weight loss but not different with groups	Significant changes HbA1c and fasting insulin but not different with groups	Significant changes in LDL and VLDL cholesterol and breast cancer markers but not different with groups		High feasibility for intervention arm (89.5% of CGM wear); high satisfaction and acceptability (80% reported that intervention is excellent)
Chekima et al,^17^ Malaysia	More reduction in weight and BMI in the intervention group	Reduction in fasting plasma glucose only in intervention group; HbA1c reduction but in both groups	In the intervention group: greater reduction in fat mass; reduction in total cholesterol; increase in HDL; reduction in total lipids to HDL ratio	Increase in mean physical activity for the intervention group	
Chekima et al,^26^ Malaysia					Rice option with low G.I. found to effect a significant lower 24-hour mean glucose; participants changed their preferences for the type of rice to the option with lower GI after seeing the effect on 24-hour mean glucose
Ahn et al,^18^ Republic of Korea	Reduction in BMI and waist circumference but not much different between groups	Reduction in HbA1c, but not much different between groups		Improvement in eating self-efficacy	
Kitazawa et al,^19^ Japan	Reduction in BMI and waist circumference	Improvement in TIR in intervention group	Reduction in diastolic blood pressure	Reduced carbohydrate intake in intervention group	

Abbreviations: T2DM, type 2 diabetes mellitus; HRQL, health-related quality of life; LDL, low-density lipoprotein; VLDL, very low–density lipoprotein; CGM, continuous glucose monitor; TIR, time in range; G.I., glycemic index; BMI, body mass index.

#### Anthropometric measures

Anthropometric measures were reported in eight studies,^15-19,21,22,24^ with significant improvements in anthropometric outcomes in five of these studies.^15-19^ Significant reductions in BMI, body mass, and waist circumference were consistently observed, with notable body mass loss occurring as early as after two weeks in one study.^16^

#### Glucose control

Nine (75%) studies^16-19,21,22,24-26^ measured markers related to glucose control. These included traditional markers such as HbA1c,^16-18,24,25^ fasting insulin,^16,17^ and fasting glucose.^16,17,19,25^ Four studies reported additional markers of insulin resistance such as HOMA-IR (homeostatic model assessment for insulin resistance).^16-19^ Indices related to glucose variability, including time in range, time above range, and time below range,^19,25^ were used to assess glucose control over time. The most recent study^19^ utilized specific measures, namely the mean amplitude of glycemic excursions and the continuous overall net glycemic action.^12^

#### Other metabolic/biochemical markers

In half of studies,^16-19,21,22^ additional metabolic/biochemical markers were reported, including cardio-metabolic risk factors,^16^ lipid profiles,^16,22^ and blood pressure.^19^ One study extended its scope to include breast cancer biomarkers.^22^ Although not statistically significant in all cases, improvements in such outcomes were reported, particularly in cardio-metabolic risk scores^16^ and lipid profiles.^17,22^

#### Behavioral change and PROMs

Six (50%) studies.^15-18,23,24^ (50%) studies assessed behavior change and PROMs, including measures of eating self-efficacy,^15^ exercise motivation,^23^ and physical activity behavior^17,18^(Table 5). Dietary behaviors, such as adherence to low glycemic index diets,^16^ and other psychosocial outcomes (health-related quality of life,^15^ depression, and insomnia)^18^ were also explored. Three studies employed an objective means to assess measures (eg, percentage of classes attended,^15^ physical and sports activities per week,^16^ metabolic equivalents of task per week)^17^. These outcomes were reportedly positively influenced by the glucose monitoring technology–based interventions, with studies reporting significant increments in self-efficacy,^15^ exercise motivation,^16^ and improvements in dietary behavior.^18^

#### Feasibility and acceptability

Feasibility, adherence, and acceptability were assessed in five studies.^20-24^ These outcomes examined user satisfaction, intervention adherence, and willingness to continue using the technology. Feasibility was rated high across studies and was measured in different ways, including by the number of glucose monitoring sensors provided and non-usage attrition,^20^ the proportion of valid days of CGM use, and the number of pre-meal hunger tracking made with CGM.^22^ Acceptability was assessed through participant satisfaction with CGM use, adherence to logging glucose measurements,^24^ satisfaction surveys addressing ease of use and difficulty participants experienced in adjusting their eating behavior based on glucose readings.^22^ Acceptability was equally rated high with participants expressing high levels of satisfaction with CGM use and indicating that they would recommend the technology to others.

### Qualitative Outcomes Reported

Qualitative data from six studies^15,20,21,23,24,26^ were synthesized, revealing five themes: (1) perceived benefits and motivations for behavior change; (2) usability and wearability; (3) feasibility and acceptability; (4) barriers and concerns; and (5) the impact on knowledge and awareness.

Perceived benefits and motivations for behavior change (theme 1): Participants found the technology to be beneficial in helping them understand how their behaviors, such as exercise, diet, and sleep, affected their glucose levels. The real-time feedback provided by the CGM technologies motivated many participants to increase their physical activity and make healthier food choices.^15,23,24,26^
Usability and wearability (theme 2): Three studies assessed the overall usability and wearability of the CGM technologies, with participants finding the devices easy to use and generally comfortable to wear.^15,20,21^ Most participants reported that CGM was painless^15,20^ and the convenience and comfort of using the devices were seen as significant advantages.^15,20,21^
Feasibility and acceptability (theme 3): Two studies assessed the acceptability and feasibility of the technology were high, with many participants expressing willingness to use the technology again in the future. Participants appreciated the continuous data provided, which they found informative and reassuring, especially in comparison to traditional glucose monitoring methods.^20,23^
Barriers and concerns (theme 4): While the general response to the glucose monitoring technology was positive, certain barriers and concerns were identified. Some participants experienced minor inconveniences in daily life, such as sleep disruptions or difficulties during activities like showering or using hot tubs.^23^ Concerns about data privacy also emerged, with participants questioning who had access to their data^20,23^ (eg, whether it was limited to the research team or could also be viewed by the technology manufacturers). Additionally, the visibility of the glucose monitoring sensor to others when worn created a degree of social discomfort for some participants.^20^
Impact on knowledge and awareness (theme 5): A significant finding from the qualitative data was also the impact of the applied glucose monitoring technology on participant knowledge and awareness of their glucose levels. Participants reported that the ability to visualize the immediate effects of food choices and physical activity on their glucose levels was particularly valuable.^15,23,24,26^

### Funding Sources and Conflicts of Interest

Apart from two studies,^15,18^ all studies disclosed funding sources, and these included academic institutions, corporate entities, and other institutional grants. The majority were funded by academic and institutional sources, while two studies received corporate sponsorship^19,25^ from companies that provided CGMs and/other logistics for the interventions.

### Critical Appraisal

The included studies were appraised using the JHEBP tool. Three studies were considered to have a high level of evidence and were rated as level I, quality B.^16,17,19^ The remaining studies were rated as level II, quality B^18,20-22,25,26^ and level III, quality B,^15,23,24^ as presented in Table 3. Several factors affected the robustness of these studies. Three of the studies employed a quasi-experimental design and there was a lack of randomization and the presence of control groups.^15,23,25^ Sample sizes were frequently small across the studies with some studies featuring as low as 13, 14, and 15 participants,^15,24,26^ reducing the statistical power and generalizability of the findings. Five studies had study durations not exceeding four weeks^18,23-26^ and only two studies^16,21^ lasted long enough for the type of outcomes that were measured to capture change.

## Discussion

The present scoping review aimed to explore studies that have used CGM or FGM technologies in an interventional capacity for adults at risk of developing T2DM. Based on the identified 12 eligible studies (published from 2016 to 2023; 1144 adult participants, of which 353 were at risk of T2DM and used glucose monitoring technologies), these technologies were used in parallel with other components, such as behavioral change support tools. Overall, these studies indicate that the application of CGM and FGM in supporting lifestyle changes to prevent T2DM among at-risk populations is still developing.

The studies in this review were conducted across eight countries and were published within the last three years, reflecting a growing interest in applying these glucose monitoring technologies beyond their established role in diabetes management. Continuous glucose monitoring has been used in T1DM management for over two decades, following its initial approval by the US FDA in 1999.^9^ Its use in T2DM management has also gained traction, especially for those on intensive insulin regimens, where tight glucose control is essential.^27^ Guidelines such as those from the UK National Institute for Health and Care Excellence for the management of adults with T2DM and the American Diabetes Association recommend CGM for insulin-treated T2DM^28^ and that diabetes devices are offered to all people with diabetes.^29^ Continuous glucose monitoring has been highly beneficial in both type 1 diabetes and insulin-treated T2DM, seeking to improve HbA1c, reduce fear of hypoglycemia, and enhance quality of life.^27,30^

This scoping review identified 12 studies on the use of the technology in individuals at T2DM risk, conducted primarily in high-income countries, such as the United States and the United Kingdom, which have a well-established track record in health care innovation and digital health technologies.^31^ Even for T1DM, where CGM has been widely explored and adopted clinically, its use remains limited outside high-income regions.^32^ This limited representation in low- and middle-income countries (LMICs) has been attributed primarily to reasons relating to socioeconomic disparities and limited access to this technology,^33^ and it is plausible that the broader implementation of CGM use for those at T2DM risk may follow a similar pattern due to such reasons/barriers. In LMICs, the adoption of CGM has been hindered by the high cost of this technology, insufficient digital infrastructure, and limited health care funding.^32^ Recent studies on the epidemiology of T2DM have revealed markedly increased prevalence rates in areas/countries across Asia, the Middle East, and Africa.^34^ Considering the rising burden of T2DM in these regions, the absence of studies in LMICs represents a critical gap. Addressing these barriers through the provision of cost-effective technologies and investment in digital infrastructure will be essential for expanding the global applicability of CGMs.

The potential for CGM to contribute to behavioral interventions targeting improved diet, increased physical activity behavior, and reduced insulin resistance is still being investigated, particularly in the context of T2DM prevention.^35^ A recent scoping review by Jospe et al^10^ considered populations with diabetes and reported that, while CGM interventions have primarily focused on diabetes management, there is a gap in research exploring its broader role in facilitating behavior change, suggesting the need for further studies to establish its effectiveness in this area. Particularly for people not living with diabetes, Oganesova et al^36^ suggested that the use of CGMs remains inconclusive and additional research is required to assess its efficacy, particularly in preventive health contexts.

Most participants in the reviewed studies were middle-aged adults with well-established risk factors for T2DM, such as obesity or prediabetes. This age selection represents a target group for T2DM prevention and is in line with existing literature which shows that together with other risk factors, transition to T2DM is heightened around middle age.^37^ For the recent increase in prevalence of youth-onset diabetes,^38^ a few studies included younger adults,^17,26^ highlighting the importance of early intervention before significant metabolic dysfunction occurs. Of note, ethnic diversity in the included studies was limited, with most focusing on white populations, thereby restricting the generalizability of findings. To ensure broader applicability, researchers should prioritize the inclusion of more diverse populations, particularly given the higher T2DM risk in certain ethnic groups. Encouraging participation from underrepresented ethnicities in countries already utilizing this technology could significantly enhance the relevance and impact of future studies.

Participants in the included studies were generally well educated, which highlights a gap in the existing research regarding the acceptability and effectiveness of such technology in populations with lower education and digital literacy, particularly given the strong link between the use of digital technology and digital literacy. For example, studies have shown that people with higher digital literacy, often linked to higher education, are more likely to adopt and effectively use health care technologies for better health outcomes.^39,40^ This emphasizes the need to explore how such digital health tools can be tailored for populations with lower digital literacy, as successful technology implementation often hinges on individuals’ ability to navigate and understand digital platforms.^41^ Focusing on people with broader socio-demographic characteristics will be important in future studies to mitigate risk of non-representative, non-inclusive, and non-generalizable samples.

The included studies primarily targeted dietary behaviors, physical activity, or a combination of both. More broadly, multi-component interventions, combining both physical activity and dietary changes, have been found to be more effective in producing sustained health improvements, particularly in weight loss. Indeed, within a targeted diabetes prevention context, multi-component interventions have been consistently shown to be more effective in diabetes prevention programs.^42,43^ For example, the Chinese Da Qing study and the Finnish Diabetes Prevention Study demonstrated that targeting both diet-related behavior and physical activity significantly reduced the incidence of T2DM compared to single-component interventions.^42,43^

The inclusion of educational components has been critical in helping participants understand how to interpret and act on the data presented by the technology. However, in many studies included in this review, the absence of educational components posed a significant barrier. Education sessions are crucial in helping participants interpret glucose-related data, which many individuals may not be as familiar with when compared to more common physiological parameters such as blood pressure and heart rate. Without this understanding, participants may struggle to act on the obtained monitoring data effectively, limiting the potential impact of these technologies. Incorporating artificial intelligence (AI) into these interventions could provide personalized feedback, predictive analytics, and tailored recommendations to enhance participants’ understanding and engagement.^44^ Relevant education approaches such as the integration of AI-driven insights, should thus be further researched and standardized to maximize the effectiveness of glucose monitoring technologies in populations at T2DM risk.

In addition to weight loss and glucose control, included studies targeted various other outcomes, including insulin sensitivity, behavioral changes, and other metabolic markers. While weight loss and improved glycemic control were in most cases found to be statistically significant, additional outcomes (eg, metabolic markers) may offer a deeper insight into the effects of the applied intervention on the overall metabolic health. For example, hyperglycemia is strongly linked to other metabolic disturbances, such as dyslipidemia and endothelial dysfunction, which significantly increase the risk of cardio-metabolic complications,^45,46^ hence these parameters could be further explored. Behavioral change and PROMs are also central to understanding the success of these interventions in promoting long-term and healthy lifestyle changes. Biological and behavioral outcomes have been extensively measured in other populations of different diabetes status, particularly those with T2DM, where they have yielded statistically significant positive results.^10^ As such, a broader selection of reported study outcomes may be beneficial to demonstrate potential impact on overall metabolic function.

Qualitative data offered insights into participants’ experiences with the CGM and FGM devices. In the six included studies with such data, the reported feedback indicated that the technology was well received, with participants finding it easy to use and integrate into their daily lives. However, concerns regarding sensor durability, comfort, and data privacy emerged. Of note, certain concerns raised on durability and comfort are related to device design and have been addressed by the progress in the relevant technologies since these studies were conducted. For example, glucose monitors that do not require any intermittent scanning are becoming more common as the technology advances. Given the importance of user experience and acceptance in interventions involving such technological elements, encouraging the integration of qualitative methods in future research should be encouraged to help refine the design of both the technology and interventions.

### Strengths and Limitations

This scoping review included a rigorous search strategy of the most common relevant databases and followed the established guidance regarding its conduct and write-up, using the JBI guidance and CONSORT for scoping reviews. We have also jointly screened all articles and conducted a critical appraisal which is not a common practice for scoping reviews.

However, certain limitations should be acknowledged, including limiting the eligibility to studies published in the English language. Additionally, unpublished studies and grey literature were not included in the review. The included studies also had limitations that may have probable effects on the strength and quality of the available evidence. Key issues included small sample sizes, a lack of diversity in participant demographics, and short study durations, all of which restrict the generalizability of findings and hinder the evaluation of long-term outcomes.

### Implications for Future Research

Future research should focus on addressing the gaps in the available evidence in this field that have been identified in the context of the present review. Larger and more diverse studies with longer follow-up periods are needed to assess the long-term effectiveness of interventions with CGM in at-risk populations. Additionally, studies should incorporate qualitative methods to better understand user experiences and to improve the design and implementation of such technology-based interventions. Further exploration of multi-component interventions, combining glucose monitoring technologies with educational, behavioral support, and AI-driven approaches, could be beneficial in optimizing the potential of CGM or FGM use within a T2DM prevention context.

## Conclusion

The evidence synthesis and findings from the present scoping review suggest that CGM has promising potential to support lifestyle and behavior changes in adults at risk of developing T2DM. While the studies reviewed provide early evidence of the role that CGM may play in improving physical activity, dietary habits, and metabolic outcomes in such adult populations, the research remains early and relatively limited; hence, longer and more robust/high-quality studies are necessary to support these initial findings which may then allow the future undertaking of a meta-analysis. Notably, research to date has already influenced policy changes, such as the recent approval by the US FDA of over-the-counter CGM systems for use by individuals without T2DM.^47^ This policy shift highlights the potential for this technology to expand beyond diabetes management into preventive health initiatives. To fully harness the potential of CGM as a tool for mitigating the risk of T2DM, future research should focus on producing more robust evidence, particularly through larger, well-powered RCTs with focus on diverse at-risk populations. With more comprehensive evidence, interventions involving CGM technologies for individuals at risk of T2DM could transition from experimental research to practical, real-world applications across the globe, potentially reshaping public health strategies for diabetes prevention.
